# A Role for Epitope Networking in Immunomodulation by Helminths

**DOI:** 10.3389/fimmu.2018.01763

**Published:** 2018-07-31

**Authors:** E. Jane Homan, Robert D. Bremel

**Affiliations:** EigenBio LLC, Madison, WI, United States

**Keywords:** helminth, immunomodulation, bioinformatics, T regulatory, networking, T-cell-exposed motifs

## Abstract

Helminth infections, by nematodes, trematodes, or cestodes, can lead to the modulation of host immune responses. This allows long-duration parasite infections and also impacts responses to co-infections. Surface, secreted, excreted, and shed proteins are thought to play a major role in modulation. A commonly reported feature of such immune modulation is the role of T regulatory (Treg) cells and IL-10. Efforts to identify helminth proteins, which cause immunomodulation, have identified candidates but not provided clarity as to a uniform mechanism driving modulation. In this study, we applied a bioinformatics systems approach, allowing us to analyze predicted T-cell epitopes of 17 helminth species and the responses to their surface proteins. In addition to major histocompatibility complex (MHC) binding, we analyzed amino acid motifs that would be recognized by T-cell receptors [T-cell-exposed motifs (TCEMs)]. All the helminth species examined have, within their surface proteins, peptides, which combine very common TCEMs with predicted high affinity binding to many human MHC alleles. This combination of features would result in large cognate T cell and a high probability of eliciting Treg responses. The TCEMs, which determine recognition by responding T-cell clones, are shared to a high degree between helminth species and with *Plasmodium falciparum* and *Mycobacterium tuberculosis*, both common co-infecting organisms. The implication of our observations is not only that Treg cells play a significant role in helminth-induced immune modulation but also that the epitope specificities of Treg responses are shared across species and genera of helminth. Hence, the immune response to a given helminth cannot be considered in isolation but rather forms part of an epitope ecosystem, or microenvironment, in which potentially immunosuppressive peptides in the helminth network via their common T-cell receptor recognition signals with T-cell epitopes in self proteins, microbiome, other helminths, and taxonomically unrelated pathogens. Such a systems approach provides a high-level view of the antigen-immune system signaling dynamics that may bias a host’s immune response to helminth infections toward immune modulation. It may indicate how helminths have evolved to select for peptides that favor long-term parasite host coexistence.

## Introduction

Parasitic helminth infections affect over 2 billion people, mostly in tropical and subtropical countries, causing a vast burden of clinical and subclinical disease (1–3). To allow them to infect their hosts for extended periods, sometimes over decades, helminths have evolved to modulate their host’s immune responses (4–6). Down-regulation of the immune response results in many infected individuals being asymptomatic carriers and thus ensures perpetuation of the parasite life cycle.

Immune modulation by helminths extends to other immune stimulants; in infected individuals the immune response to secondary parasite infections, to other pathogens, and to vaccines is also down-regulated (7–11). While epidemiologic and socioeconomic factors also contribute to the prevalence of co-infections, helminth-induced immune modulation may predispose to infection by *Mycobacterium tuberculosis* or malaria (7, 12–14). A number of helminth infections have been associated with increased risks of cancer. The oriental liver flukes, *Clonorchis sinensis* and *Opisthorchis viverrini*, are risk factors for development of cholangiocarcinoma, typically many decades after initial infection (15). Infection with *Schistosoma haematobium* is associated with an increased risk of bladder carcinoma (16). Intestinal helminths have been thought to reduce risk of *Helicobacter pylori* associated adenocarcinoma (17), but *Opisthorchis* flukes may serve as risk factor by vectoring *H. pylori* and thus increasing associated cancers (18).

Helminth immune modulation has some beneficial effects as allergies, and inflammatory and autoimmune diseases are less common in populations infected with helminths (11, 19–21). Treatment with anthelminthics removes this effect and is reported to increase the incidence of inflammatory, autoimmune and allergic diseases (22, 23). The observation that helminth infection modulates inflammation and allergic responses has raised interest in the use of helminth infections, helminth extracts, or recombinant helminth-derived proteins as therapeutic interventions (24–27).

The mechanisms of immunomodulation arising from helminth infections have been extensively researched and are the subject of a large body of literature and many authoritative reviews (4, 11, 27–29). Among the many reports of possible modes of action (30, 31), two common themes emerge. First, certain groups of proteins appear to play a key role in bringing about changes in the host immune response. This includes proteins, which are secreted or excreted (32–34), proteins that are present in outer surface tegument or gastrointestinal surfaces, or proteins that are continually shed into the environs of the worm, either in isolation or as components of extracellular vesicles (35, 36). Extracts of secreted and surface proteins have been shown experimentally to elicit some of the immunomodulatory effects (32, 34). This has been extended to testing a number of individual proteins and identifying several proteins that can affect immune function (24, 28, 31, 37). The second unifying theme is that T regulatory (Treg) cells, and induction of IL-10, play a central role in helminth-induced host immune modulation (38–44), whether by classical Foxp3+ CD4 cells or other IL-10 secreting populations of CD4+ or CD8+ cells (45–50).

Helminth infections are not uniformly immunosuppressive. Protective immunity does emerge over time and provides the impetus for research toward vaccines (1, 51, 52), although reinfection may follow anthelmintic treatment (53, 54). Allergic responses to some helminths also occur and may also predispose to asthma (55, 56).

Studies of helminth immune modulation have largely focused on how cytokine-mediated effector mechanisms may impact the immune response. However, they have not addressed the question of whether the initial signaling in immune recognition of the parasite causes components of the immune response to be biased toward a suppressive or regulatory pathway.

In this study, we use a computational systems approach to evaluate the initial signal recognition patterns between helminth and host T cells. We evaluate the complete proteomes of 17 representative helminths and three reported co-infections based on the pattern of amino acids that would be exposed to T cells, which determine the interaction of parasite antigen and T cells. Essentially, we are attempting to see the helminth antigens as a T-cell receptor would see them, based on the minimal differentiating signal patterns from major histocompatibility complex (MHC)-bound helminth peptides that would be exposed from the MHC histotope. We have previously examined such interactions between T-cell receptor and amino acid motifs in a variety of bacterial pathogens and commensals and found distinct patterns that appear to relate to the pathogenesis of the organisms (57).

A single T-cell does not recognize, or respond to, a whole helminth; it instead engages with the few amino acids in a bound peptide MHC (pMHC) protruding from a MHC histotope, which is determined by the host human leucocyte allele (HLA) genotype. Protein antigens are processed by antigen-presenting cells (APCs) and presented to T cells as peptides bound in the groove of MHC molecules. The bound peptides (8–11 amino acids for MHC I and 13–22 amino acids for MHC II) comprise inward facing amino acids which determine peptide binding in the groove pockets [the groove-exposed motif (GEM) or pocket positions]. The intervening amino acids protrude outwards from the MHC surface or histotope and are thus exposed to the T-cell receptor, determining binding to that cognate receptor (58, 59). We refer to these amino acid motifs as the T-cell-exposed motif (TCEM). In MHC I-bound peptides, the TCEM comprises a continuous pentamer comprising positions 4, 5, 6, 7, and 8 of a nonamer, whereas in MHC II-bound peptides, the TCEM is a discontinuous pentamer in two possible recognition patterns, denoted here as TCEM IIA and IIB and comprised amino acid positions ~2,3~5~7,8~ or −1~~3~5~7,8~, respectively (where ~ is the intervening positions), relative to the central nonamer core of a longer bound peptide (60, 61). Hence, the signal that determines the outcome of the interaction with the cognate T cell is determined by the pentameric TCEM motif. This enables T cells to be polyspecific, responding to the same TCEM recognition signal, even though the MHC-bound peptide may be derived from antigens of different sources (62). The duration of TCEM signaling is determined by the stability of the pMHC complex and thus the dwell time of the peptide in the MHC groove, a function of the amino acids making up the MHC GEM. The frequency of signaling is determined by the size of the cognate T-cell population encountering the signal, which in turn is influenced by the frequency of occurrence of that TCEM in self proteins, commensal microbiome, and other pathogens.

The proteins of the human microbiome, especially the gastrointestinal microbiome, the human proteome, and the immunoglobulin repertoire are also continually processed by APCs and presented to T cells (63, 64). In examining the immunoglobulinome, it emerged that there is a frequency hierarchy of TCEM. This includes, at one extreme, common motifs found in most immunoglobulin variable regions. These are not limited to motifs encoded by the germline but also include motifs produced by somatic mutation. At the other extreme, very rare motifs are encountered only once in several million B-cell clones (65, 66). This frequency distribution is representative of that in the rest of the human proteome and gastrointestinal microbiome (57). The frequency of appearance of a TCEM determines the frequency of stimulation of a clone or clones of cognate T cells. Hence, more common motifs would be expected to beget larger cognate T-cell populations than rare motifs. A highly bound peptide that recruits a large T-cell population and a high frequency of signal is more likely to generate a suppressive or Treg response (67–72). The human immunoglobulinome comprises the greatest diversity of motifs relative to the rest of the human proteome and microbiome and undergoes considerably more turnover. We use the immunoglobulinome variable regions as a reference scale of TCEM motif frequency (65). Here, we show that a feature of helminths is their content of common TCEM that, when in the context of a binding GEM, is potentially immunosuppressive and to which the infected host is chronically exposed.

Helminth parasites have large genomes and complex life cycles (73, 74). Considerable progress has been made in helminth genome sequencing, but many proteins remain unannotated, and transcriptomic studies that relate proteins to life stage, while more advanced for a few helminths (75–79) are mostly incomplete. With these limitations, we cannot define an exact combination of T-cell epitope modulation signals functioning within the human host, but the results we present are indicative of the complexity of potential T-cell epitope interactions within and among helminth species and with potential co-infections. Such a systems approach provides a high-level view of the antigen-immune system signaling dynamics that may bias a host immune response to helminth infections toward immune modulation and may suggest approaches to develop a further understanding of the host:parasite interrelationship.

## Materials and Methods

### Helminth Proteomes

Protein FASTA files for 17 complete helminth proteomes were downloaded from Wormbase (www.wormbase.org) (80) as shown in Table 1. All infect humans; most have human as their primary definitive host; some (e.g., *Echinococcus granulosus, Trichinella spiralis, Fasciola hepatica*, and *Loa loa* infect humans as an accidental zoonotic host). Proteomes identified and curated by different originating laboratories varied in the size criteria applied for inclusion as did criteria for inclusion of multiple splice variants. Proteins under 50 amino acids were excluded from our analysis as end effects of the sliding analysis windows preclude making meaningful comparisons to larger proteins.

**Table 1:** Helminth proteomes analyzed.

Class	Parasite evaluated	Proteome size^a^	15-mer peptides	Secreted and surface proteins selected for complete analysis^b^	Secreted and surface proteins selected as percentage of proteome	Project number on http://parasite.wormbase.org	Reference
Nematodes	*Ancylostoma ceylanicum*	36,687	9,508,454	3,266	8.9	prjna231479	(75)
	*Ancylostoma duodenale*	27,485	5,447,645	713	2.6	prjna72581	
	*Necator americanus*	19,153	4,868,515	737	3.8	prjna72135	(81)
	*Ascaris lumbricoides*	23,604	6,833,958	1,064	4.5	prjeb4950	
	*Brugia malayi*	11,021	6,125,076	853	7.7	prjna10729	(82)
	*Loa loa*	12,473	4,931,854	606	4.9	prjna246086	(83)
	*Onchocerca volvulus*	12,117	4,873,818	736	6.1	prjeb513	(84)
	*Wucheria bancrofti*	13,058	4,140,788	589	4.5	prjeb536	
	*Trichinella spiralis*	14,737	8,879,076	1,567	10.6	prjna257433	(85)
	*Trichuris trichuria*	9,650	4,050,951	522	5.4	prjeb535	(86)

Trematodes	*Clonorchis sinensis*	13,634	7,029,209	933	6.8	prjda72781	(87)
	*Opisthorchis viverrini*	16,356	6,848,490	874	5.3	prjna222628	(88)
	*Fasciola hepatica*	22,676	5,851,887	670	3.0	prjeb6687	(89)
	*Schistosoma mansoni*	10,772	5,443,618	629	5.8	prjea36577	(90, 91)

Cestodes	*Diphylobothrium latum*	19,966	3,872,702	529	2.6	prjeb1206	
	*Echinococcus granulosus*	10,245	4,932,446	804	7.8	prjeb121	(92)
	*Taenia solium*	12,481	4,992,618	724	5.8	prjna170813	(92)

*^a^T-cell-exposed motif (TCEM) extraction and ranking were completed on the whole proteome*.

*^b^Proteins for detailed analysis were selected from the proteome based on the presence of a transmembrane domain and/or signal peptide, which are in the top 5% of content of high frequency TCEM (those motifs more common than 1 in 1,024 clones), or which contain TCEM in any pattern (TCEM I, IIA, or IIB)*.

### Comparative Proteomes

The proteins of the human immunoglobulinome, comprising the variable region sequences of 40 million distinct immunoglobulins, were assembled as previously described, supplemented with sequences from clinical samples and other published immunoglobulin sequences, and the TCEM frequency distributions were precomputed as previously described (61, 66, 93). Examination of the frequency of occurrence of TCEM in the large immunoglobulin dataset allows us to assign a frequency category (FC) to each TCEM motif as it occurs in the immunoglobulinome clonotypes. Each motif in each recognition pattern (TCEM I for MHC I and TCEM IIA and IIB for MHC II) is assigned a log base 2 FC based on whether it occurs in half (FC1), 1 in 4 (FC2), and 1 in 8 (FC3) immunoglobulin variable regions, up to FC23, which represents 1 in 8.39 million (2^23^) clonotypes, or approximately one T-cell clone in the entire repertoire that a single human carries. A further category of FC24 designates those motifs not yet encountered in immunoglobulin variable regions analyzed to date. The mean frequency in the human immunoglobulinome is FC10, a motif which is found in 1 in 1,024 (2^10^) variable regions. Motifs of FC1-3 are referred to as very high frequency and those of FC10 or less as high frequency.

The human proteome, including all known isoforms of each protein (totaling 88,000 proteins), but excluding immunoglobulin sequences, was assembled from Uniprot (www.uniprot.org) and its TCEMs precomputed. The proteomes of constituents of the GI microbiome were assembled and precomputed as previously described (57). This database comprised 67 bacterial species.

Proteomes of reference sequences of *Plasmodium falciparum* (3D7), *M. tuberculosis* (H37Rv), and *H. pylor*i (26695) were downloaded from EupathDb (http://eupathdb.org) and Patric (https://www.patricbrc.org/) (94, 95). These proteomes were screened in their entirety for high frequency TCEM and not filtered by SP and transmembrane helix (TMH) proteins.

### Determination of TCEMs and Predicted MHC Binding

T-cell-exposed motif patterns were extracted from the complete proteomes and ranked as previously described for each of three recognition patterns (61, 65). Frequencies of motif occurrence were determined with respect to the immunoglobulinome, the human proteome, and the gastrointestinal microbiome (57). MHC binding was predicted using neural network ensembles trained to the LN ic50 (96) for approximately 230,000 binding reactions using the neural platform of JMP^®^ (SAS Institute). This is an updated version of a system described previously (61). Predicted MHC binding was computed for each sequential 9-mer and 15-mer peptides in selected protein sets, wherein the predicted binding affinity to 37 MHC I and 28 MHC II alleles was determined. To estimate population behavior comprising multiple alleles with varying affinities for any peptide, the LN ic50 binding data estimates were transformed and standardized to a zero mean unit variance within each protein using a Johnson Sb distribution (97). This transformation enables the combination of binding results from different alleles, which is akin to using the principle of the additivity of variance commonly used in quantitative genetics (98, 99). The probability of cleavage of each protein by human cathepsin B, L, or S was determined. Both binding affinity and cleavage predictions were accomplished using previously described methods by neural network predictors based on principal component analysis of amino acid physical properties (100).

### Suppressive Indices

To derive an indicator of the probability of a particular peptide generating a Treg response, we combined the prediction of standardized MHC binding and the frequency of occurrence of the TCEM. The combination of these two parameters provides a metric that is directly related to the probability that a collision between a cognate T cell and a pMHC on the surface of an APC will occur. For each peptide, where the standardized predicted binding affinity is greater than the mean for the protein and allele combination, the predicted standardized affinity of each allele (A) is weighted by its cognate encounter number (nA × FC^16-FC^) and the sum that product provides the suppressive index. Thus, the final suppressive index is a composite metric of affinity and how often cognate T-cell encounters will occur in the human population (Figure S1 in Supplementary Material).

The suppressive index is therefore an indicator of the predicted response of the human population of diverse immunogenetics; the response of any particular HLA allele will vary around this mean index. Hence the suppressive index, as computed here, does not distinguish the differences in modulation, which may arise between different ethnic or immunogenetic population subsets or between individual immunogenetically distinct hosts.

Bacteria and viruses, with small proteomes, typically comprise peptides with suppressive indices of up to a few hundred and usually under about 8,000, rare motifs have indices of 20,000–50,000 (data not shown). Only exceptional proteins in bacteria and viruses have motifs with indices >200,000. A peptide scoring a suppressive index over 1 million is thus extremely rare and would only be possible with an extremely common motif (FC1-3) that binds to almost all human alleles (in either frames IIA and IIB or MHC I A and B).

### Selection of Proteins Comprising Potential Suppressor Motifs

Given the large size of each helminth proteome, we focused attention particularly on proteins of the secreted and surface proteins and on those most likely to elicit a suppressive response. To this end, all proteins with signal peptides and/or transmembrane helices in an organism proteome were selected. To identify transmembrane regions and signal peptides in the primary amino acid sequence of proteins, the online resource Phobius http://phobius.sbc.su.se/was used. This combines the two types of domain identification tools generally used in genome annotation in one resource (101). Signal peptides were predicted for eukaryotic organisms.

From these, in order to focus on the likely immunomodulatory protein candidates, two subsets of proteins were then compiled from each species based on the following criteria: the proteins having the highest content of common TCEM (less than or equal to FC 10) per 100 amino acid length were selected; and those proteins having any TCEM (TCEM I, IIA, or IIB) of FC1-3 (motifs occurring at least as frequently as in one in eight immunoglobulin variable regions). These subsets were combined, duplicates were eliminated, and a full computation of predicted MHC binding and cathepsin excision sites was completed. This comprised from 2.5 to 10% of each proteome. All peptides for each protein in each species set (15-mer for MHC II and 9-mer for MHC I) were ranked by suppressive index. Any peptide having a suppressive index over 300,000 and its source protein were identified.

### Proteins Reported to Be Associated With Immunomodulation

A set of sequences for proteins previously reported to be associated with immunomodulation (24, 28, 37) was assembled, using Reference sequences from GenBank. These proteins are listed in Table S1 in Supplementary Material.

### Statistical Analysis

All data processing, pattern analysis, and statistical analysis were done with JMP^®^ v13 from SAS Institute (Cary, NC, USA).

## Results

Analyses were conducted for all three TCEM recognition patterns in the complete proteomes. To conserve space, in some cases, we show data for TCEM IIA in the body of the paper and provide corresponding TCEM I and TCEM IIB data in Supplementary Tables. Very similar results are seen for each of the TCEM patterns.

### TCEM Characteristics of the Entire Organism Proteome

#### Sharing of TCEMs Is Common

For each TCEM pentameric motif pattern, the maximum possible configurations of the 20 amino acid is 3.2 million (20^5^). All possible motifs are found in the immunoglobulinome. The human proteome and GI microbiome comprise approximately 75 and 91%, respectively, of the possible motifs in each recognition pattern.

Table 2 shows the content the TCEM IIA motifs in the helminth species studied and the degree of sharing of motifs (Table S2 in Supplementary Material shows additional motif patterns TCEM I and IIB). The repertoire of each helminth comprises from approximately 53 to 71% of the total possible motifs in each TCEM pattern, indicating the overlap of repertoire with the immunoglobulinome. Among all 17 species of helminth, >96% of all possible TCEMs are collectively represented.

**Table 2:** Sharing of total T-cell-exposed motif (TCEM) IIA between helminths, gastrointestinal microbiome, and human proteome.

	Total TCEM IIA	TCEM IIA as percentage of 3.2 million	TCEM IIA shared with GI microbiome	Percentage of TCEM IIA shared with GI microbiome	TCEM IIA shared with human proteome	Percentage of TCEM IIA shared with human proteome	TCEM IIA unique to helminth species	Unique TCEM IIA as percentage of species total
Immunoglobulinome	3,200,000	100.00						
GI microbiome	2,921,445	91.30						
Human proteome #1	2,412,699	75.40						
Total motifs in 17 helminths	3,101,990	96.94						
TCEM IIA common to all 17 species	418,120							
*Ancylostoma ceylanicum*	2,281,190	71.29	2,193,019	96.13	1,931,093	84.65	13,051	0.57
*Ancylostoma duodenale*	1,844,757	57.65	1,790,685	97.07	1,609,132	87.23	4,032	0.22
*Necator americanus*	1,845,715	57.68	1,792,570	97.12	1,611,216	87.29	5,294	0.29
*Ascaris lumbricoides*	2,075,222	64.85	2,005,287	96.63	1,783,734	85.95	13,400	0.65
*Brugia malayi*	1,835,074	57.35	1,780,913	97.05	1,595,941	86.97	2,370	0.13
*Loa loa*	1,796,988	56.16	1,746,152	97.17	1,569,747	87.35	3,397	0.19
*Onchocerca volvulus*	1,855,127	57.97	1,800,210	97.04	1,610,305	86.80	5,015	0.27
*Wucheria bancrofti*	1,737,656	54.30	1,689,344	97.22	1,519,185	87.43	1,925	0.11
*Trichinella spiralis*	1,873,959	58.56	1,811,550	96.67	1,622,466	86.58	12,512	0.67
*Trichuris trichura*	1,716,725	53.65	1,669,456	97.25	1,507,130	87.79	9,275	0.54
*Clonorchis sinensis*	2,079,755	64.99	2,006,177	96.46	1,792,125	86.17	7,740	0.37
*Opisthorchis viverrini*	2,032,485	63.52	1,962,333	96.55	1,758,940	86.54	6,589	0.32
*Fasciola hepatica*	1,918,592	59.96	1,855,938	96.73	1,670,778	87.08	10,851	0.57
*Schistosoma mansoni*	1,822,979	56.97	1,767,657	96.97	1,586,723	87.04	9,105	0.50
*Diphyllobothrium latum*	1,592,066	49.75	1,551,677	97.46	1,417,136	89.01	6,647	0.42
*Echinococcus granulosus*	1,788,474	55.89	1,735,938	97.06	1,575,786	88.11	4,636	0.26
*Taenia solium*	1,801,611	56.30	1,748,550	97.05	1,585,759	88.02	5,002	0.28

#### Sharing Between Helminth Species

**Figure 1:**
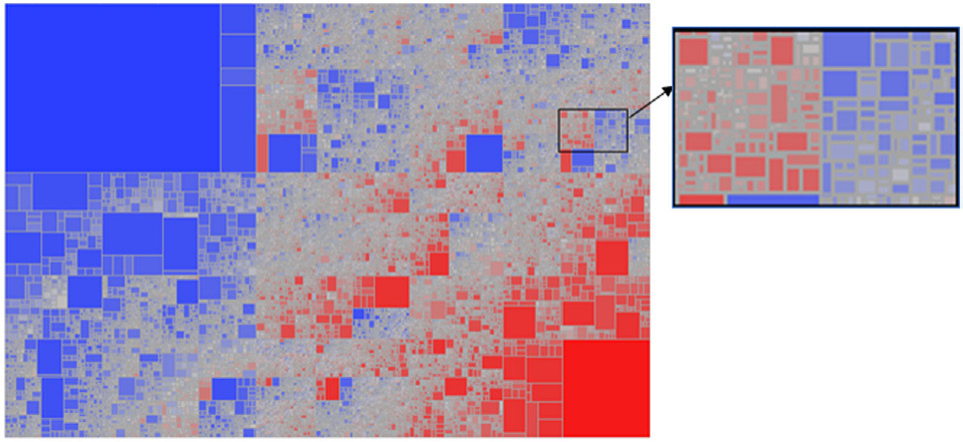
Treemap showing the complexity of sharing patterns of T-cell-exposed motifs (TCEMs) (shown for TCEM IIA) among the 17 helminth species analyzed. A total of 112,225 unique sharing patterns were identified. The sharing pattern refers to the combination of the 17 species of helminths that carries that motif. Some sharing patterns comprised a single TCEM motif represented by a very small tile; other sharing patterns comprise large groups of shared motifs and are shown by large tiles. The tiles are colored from highly shared motif groups (blue) to minimally shared motif groups (red). The large blue box at top left represents the TCEM IIA common to all 17 species; the red box at bottom right represents the TCEM IIA absent from all species. The intervening tiles each have a unique combination of motifs and species sharing pattern. The expanded high resolution cutout provides an appreciation of the number and complexity of different patterns embedded in the lower resolution picture.

Given the breadth of the TCEM repertoires of each species of helminth there is inevitably overlap in repertoires between the species. Overall ~415,000 TCEM motifs of each pattern are present in all of the 17 species analyzed. The remaining ~2.6 million motifs are shared in varying combinations among the species. Figure 1 shows the motif sharing patterns. Each tile represents a unique combination of sharing of the 3.2 million TCEM IIA motifs among the 17 species, in all comprising 112,225 different motif sharing combinations. This indicates the complexity of the potential T-cell epitope sharing and cross-reactivities between species.

Each helminth species has a small set of motifs, less than 0.6%, that are unique to that species relative to the other 16 species (last column Table 2). The exact number of these motifs is unlikely to be significant given the different approaches to sequencing and curation adopted for each species, but it is notable that each species does have a unique signature. However, it must be recognized that such unique motifs are overlapped with amino acids comprising non-unique motifs, so their utility in directing a unique immune response is limited.

#### Sharing of TCEM Between Helminths and Co-Infecting Pathogens

Three unrelated pathogens cited as common co-infections with heminthiases were selected for comparison. The extent of TCEM IIA sharing with *P. falciparum, M. tuberculosis*, and *H. pylori* is shown in Table S3 in Supplementary Material. Across all species of helminth, approximately 50% of TCEM have matches in *P. falciparum*, 28% have matches in *M. tuberculosis*, and 16% have matches in *H. pylori*.

### Distribution of High-Frequency TCEMs Within the Helminth Proteome

The frequency of occurrence of TCEMs in each helminth proteome was compared to their frequency in the immunoglobulinome. There is no significant difference in the distribution of motifs found in helminths among the frequency categories observed in the immunoglobulinome (correlation coefficients all >0.9); those motifs that are common in immunoglobulins are commonly found in helminths and rare immunoglobulin motifs are also rare in helminths.

**Figure 2:**
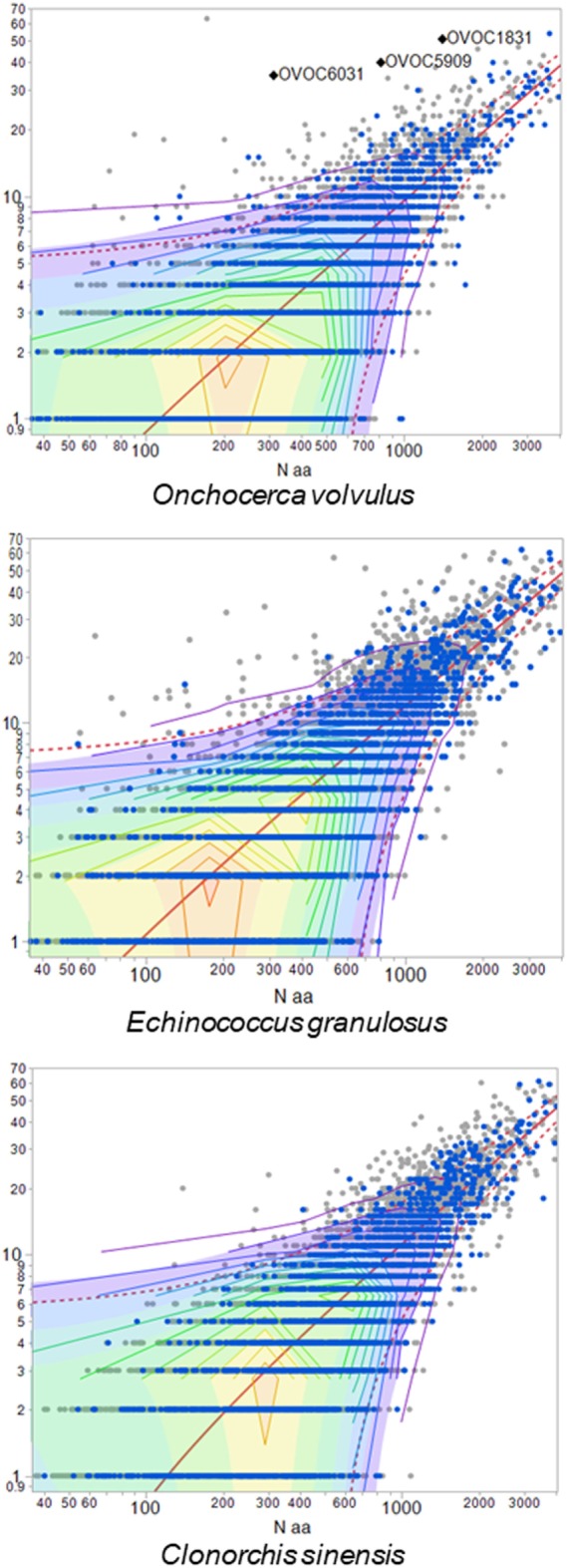
Proteins with high content of common T-cell-exposed motif (TCEM). Each dot represents one protein of the species indicated. *X*-axis shows size of the protein (log_10_ amino acid number). *Y*-axis shows number of TCEM IIA motifs of higher than FC10, i.e., occurring more often than the mean in an immunoglobulinome reference database. Proteins shown in blue are secreted and surface proteins. Three example *Onchocerca volvulus* proteins are labeled, and sequences for these provided in Figure S3 in Supplementary Material.

We then examined the distribution of high frequency TCEM in the proteins of each helminth proteome to evaluate whether particular proteins might be contributing more to an immunosuppressive response. Figure 2 shows examples of the distribution of proteins based on their content of TCEM of FC10 or lower, i.e., those motifs more common than the mean frequency in the immunoglobulin reference database. Each protein is plotted according to its size and the number of the frequent motifs. Comparable plots for the other helminths, and the three comparative co-infections, are provided in Figure S2 in Supplementary Material. While the distribution is not dissimilar from that seen in many other pathogens (57), the scatter of outlier proteins with a high content of frequent or rare TCEM is distinctive. As shown in Figure 2, the outliers are not proteins with signal peptides or transmembrane domains. Having a high content of high frequency TCEM motifs would be an indicator that a protein may have more immunosuppressive peptides, if such peptides are appropriately processed in APC and bind to MHC. On closer examination, the outliers were found to be proteins which have a high content of repetitive residues, either homorepeats of amino acids or repeats of simple amino acid motifs. Homorepeats of L, S, G, T, and Y emerge as frequent FC categories while those homorepeats of M, C, H, Q, and W are rare motifs in immunoglobulins. Figure S3 in Supplementary Material shows the sequences of those *Onchocerca volvulus* examples labeled in Figure 2, which are examples of proteins comprising amino acid repeats. *L. loa* is particularly striking with respect to the number of proteins with extended homorepeats that show as having a high content of common TCEM (Figure S1 in Supplementary Material).

### TCEM Characteristics of Surface and TMH Proteins

#### Sharing of TCEM Associated With High Suppressive Index Between Helminth Species

Given the focus in the literature on the secreted, surface, and shed proteins as potential contributors to immunomodulation (32–35), we elected to focus further analysis on the subset of proteins which have a signal peptide, one or more transmembrane helices or both, along with the highest frequency TCEM. For each helminth species, the top 2.5–10% of protein with respect to their likelihood of comprising suppressive motifs were analyzed, comprising 15,816 proteins overall (Table 1). The number of peptides with extremely high suppressive indices over 300,000 was determined and included 903 peptides with high MHC I suppressive indices, of which 627 were unique, and 1,377 peptides with high MHC II suppressive indices, of which 977 were unique. Such highly suppressive index peptides were found in all the helminth species examined. Table 3 shows the counts by species and suppressive index groupings. Among these, 70 proteins were identified containing more than one high suppressive index peptide at separate positions. Given the differing criteria in helminth proteome assembly, and the criteria imposed for the selection of analysis of a secreted or surface subset of proteins, the absolute numbers shown are not of particular significance except to indicate that such high indices are quite common findings in helminths.

**Table 3:** Content of extremely high suppressive index peptides in secreted and surface proteins, showing peptides with suppressive index over 300,000.

	Major histocompatibility complex (MHC) I	MHC II
*Ancylostoma ceylanicum*	196	322
*Ancylostoma duodenale*	36	72
*Necator americanus*	32	63
*Ascaris lumbricoides*	81	83
*Brugia malayi*	56	72
*Loa loa*	32	56
*Onchocerca volvulus*	46	65
*Wucheria bancrofti*	34	48
*Trichinella spiralis*	71	150
*Trichuris trichuria*	20	64
*Clonorchis sinensis*	70	85
*Opisthorchis viverrini*	61	101
*Fasciola hepatica*	8	17
*Schistosoma mansoni*	30	58
*Diphylobothrium latum*	1	6
*Echinococcus granulosus*	68	62
*Taenia solium*	61	53

Total	903	1,377

**Figure 3:**
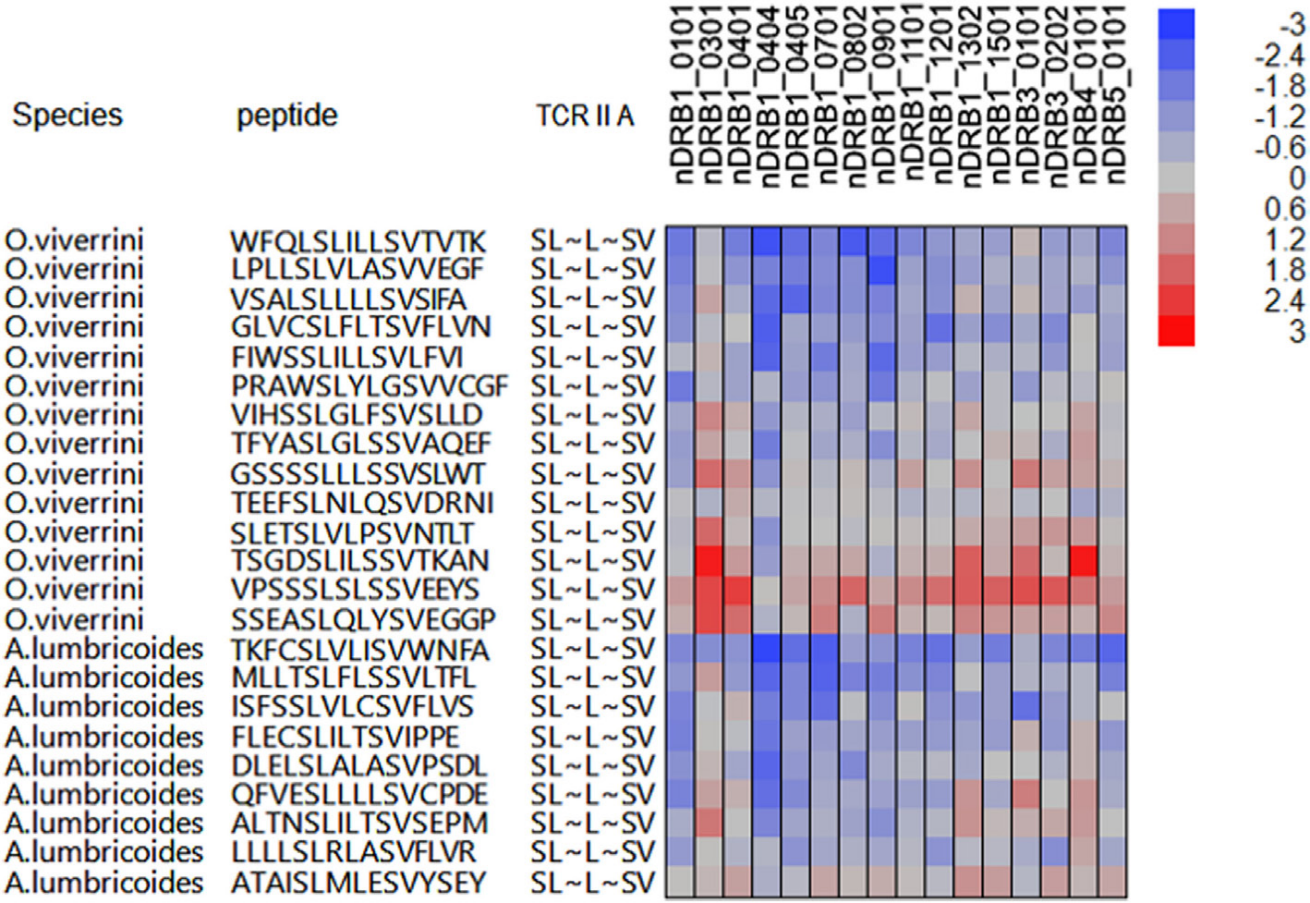
Example of peptides of *Opisthorchis viverrini* and *Ascaris lumbricoides*, which have shared T-cell-exposed motifs (TCEMs) but in the context of differing groove-exposed motifs. *X*-axis of cell plot indicates multiple different major histocompatibility complex (MHC) II DRB alleles. Coloration of the cell plot indicates predicted binding affinity in standard deviation units below the mean for that protein. Blue indicates high binding affinity; red indicates low affinity. The peptides represent a range of suppressive indices: *O. viverrini* ranges from 621,829 to 20,788 and *A. lumbricoides* from 371,273 to 36,698 based on binding to all MHC II alleles evaluated. As the TCEM motif SL~L~LV is a common motif (FC3), these are all relatively high indices.

Several helminths have peptides of high suppressive index, which comprise shared TCEM motifs, but in association with the same or different flanking peptides. Not surprisingly, the same flanking sequences are found in closely related helminths, such as *Clonorchis* and *Opisthorchis* as well as in proteins with multiple different transcripts, as shown for *Ancylostoma ceylanicum*. It should also be noted however that the same TCEM motifs may occur in the same parasites in the context of flanking peptides which are not high affinity binders. Depending on their binding affinity, these would have a shorter or even a transitory dwell time within the MHC groove. However, to the degree they may attract cognate Treg cells whose phenotype has already been determined, they may reinforce Treg signaling. Figure 3 shows an example of peptides from *O. viverrini* and *A. lumbricoides* each comprising the same FC3 TCEM IIA motif SL~L~LV but within an array of peptides, which show different predicted MHC II binding affinities. It also indicates how binding may vary between alleles to generate different outcomes in different hosts.

#### Sharing of TCEM Associated With High Suppressive Index Between Helminths and Co-Infections

**Figure 4:**
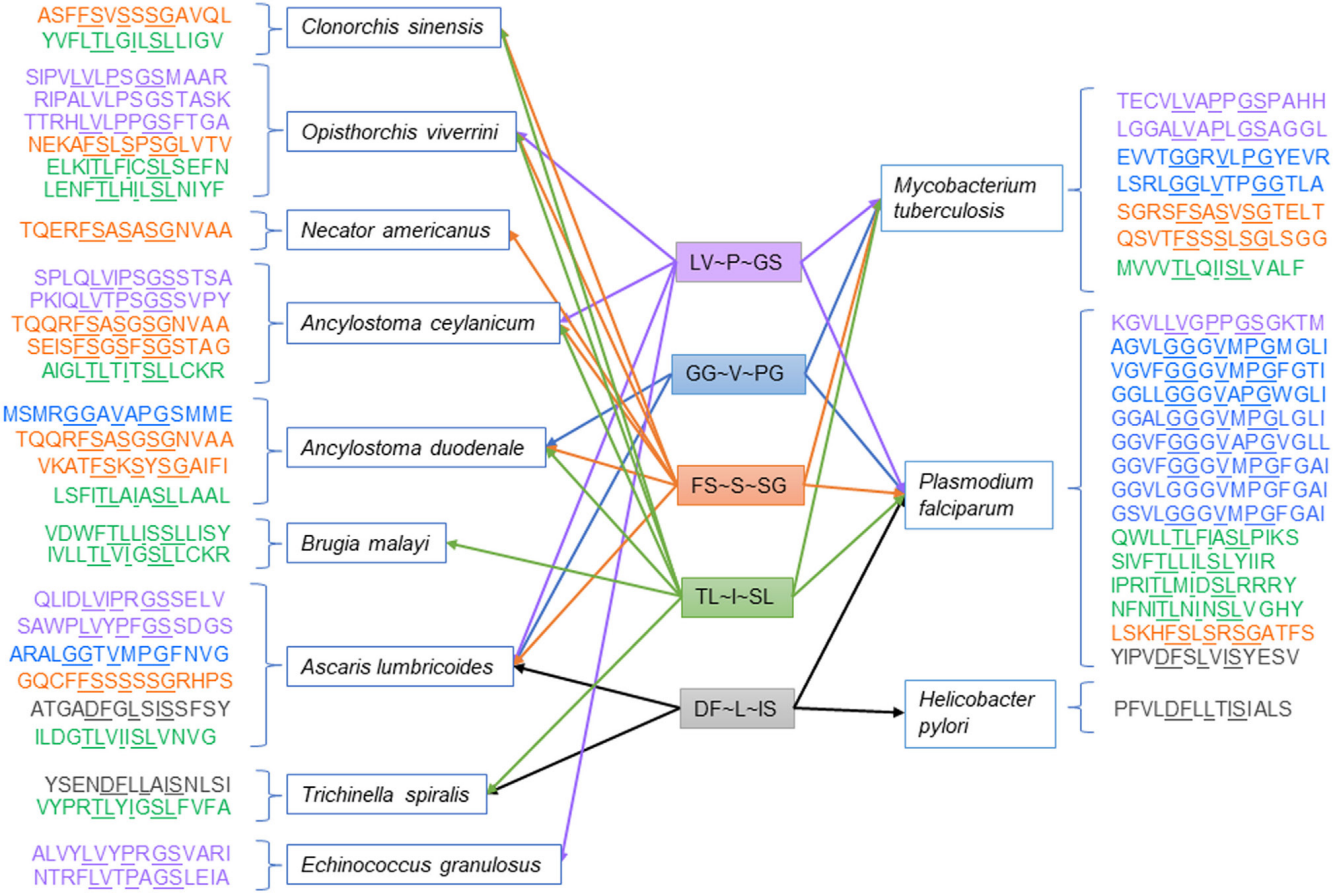
Illustration of networking among high suppressive index T-cell-exposed motifs in helminths and selected co-infections.

High-frequency TCEM associated with high suppressive indices was also shared between helminths and the three co-infecting pathogens examined. Table 4 shows the count of shared motifs and the TCEM associated with highest suppressive indices in helminths and their matches in the three co-infecting agents. Shared highly suppressive motifs were found particularly for *M. tuberculosis* and *P. falciparum*. We have focused on those with suppressive indices over 300,000. Figure 4 shows a conceptual model for how such T-cell networking among multiple helminths and co-infections may occur, based on a small illustrative group of five TCEM IIA motifs, which would each engage a finite number of T-cell clonotypes.

**Table 4:** Sharing of helminth T-cell-exposed motifs (TCEMs) associated with extremely high suppressive index motifs with selected co-infections.

	Suppressive indices >300,000								
	*Mycobacterium tuberculosi*s motifs shared			*Plasmodium falciparum* motifs shared			*Helicobacter pylori* motifs shared		
		
	TCEM I	TCEM IIA	TCEM IIB	TCEM I	TCEM IIA	TCEM IIB	TCEM I	TCEM IIA	TCEM IIB
*Ancylostoma ceylanicum*	69	51	49	90	47	57	0	0	2
*Ancylostoma duodenale*	15	10	13	21	12	15	0	1	0
*Necator americanus*	9	14	8	20	14	14	0	0	1
*Ascaris lumbricoides*	21	24	6	41	26	14	0	2	1
*Brugia malayi*	16	13	5	29	16	14	0	1	1
*Loa loa*	8	7	9	18	9	8	0	1	1
*Onchocerca volvulus*	14	10	4	24	9	12	0	1	0
*Wucheria bancrofti*	9	6	7	17	7	11	0	1	0
*Trichinella spiralis*	15	17	12	32	40	34	0	10	0
*Trichuris trichuria*	6	11	10	5	11	18	0	1	0
*Clonorchis sinensis*	24	14	8	37	12	22	0	0	1
*Opisthorchis viverrini*	20	22	10	35	21	28	0	1	3
*Fasciola hepatica*	4	8	4	7	5	3	0	0	0
*Schistosoma mansoni*	7	8	6	17	13	8	0	2	1
*Diphyllobothrium latum*	0	0	2	0	0	4	0	0	0
*Echinococcus granulosus*	25	10	13	34	5	18	0	0	3
*Taenia solium*	26	9	7	33	9	13	0	0	1

#### Proteins Previously Reported to Be Associated With Immunomodulation

Analysis of suppressive indices in 21 proteins reported to be associated with immunomodulation (Table S1 in Supplementary Material) identified only one, *T. spiralis* p53 with a peptide having a suppressive index over 300,000; the 15-mer at index position 456 has a suppressive index of 305,422, driven by the motif AV~Y~AR, which is found in 25% of all immunoglobulin variable regions. Suppressive indices of the other proteins are noted in Table S1 in Supplementary Material. Several proteins have suppressive indices in the range of 20,000–80,000.

## Discussion

Reports of immunomodulation by helminths have led to a search for individual proteins, which may cause this effect. A comprehensive explanation of helminth associated immunomodulation has been elusive, but a recurring theme is that Treg cells play a central role (38, 40). In the present analysis, we apply a systems approach examining the potential magnitude of networking of T-cell responses between parasites, host, and co-infecting microrganisms and offering a conceptual model, which may shed new light on the immunomodulation phenomenon. The goal of this study was not to identify a definitive set of peptides that are solely responsible for immunomodulation, but rather to highlight potential interplay of T-cell epitopes in an ecosystem in which helminths are present, and also to know how such networking may contribute to immunomodulation of the host.

Our study has several important limitations. Both annotation of helminth proteomes and transcriptomic studies of helminths infecting their hosts are incomplete and challenging (102, 103). Indeed, we do not know in which life stage of the parasite, many proteins are expressed or in which host microenvironment they may have an effect. Thus, we cannot anticipate the quantitative or temporal impact of each protein. In a highly expressed protein, a suppressive motif may be multiplied many times or a moderately suppressive peptide motif may carry more weight if that protein is highly upregulated. Each parasite proteome is large, and to facilitate analysis, we selected certain subsets (surface and secreted proteins) for more detailed evaluation. For most of the helminths studied, only a single proteome is available. We only studied one isolate or reference proteome for each organism, both helminth and the three selected co-infecting pathogens. Polymorphisms of proteins between isolates based on single amino acid changes are sufficient to change MHC binding of peptides and the TCEM presented (104). Furthermore, single amino acid changes impact T-cell interactions across up to 46 potential binding peptides, by changing any of cathepsin processing, MHC binding, or the TCEMs presented (65). Hence, different isolates will have somewhat different networking patterns, but the overall conceptual model will apply.

The starting point for such a networking model is the premise that a T-cell receptor is agnostic as to the source of the amino acid motif of a bound peptide, which the TCR engages in the context of the MHC histotope. A cognate TCR engages a particular pentameric motif whether that occurs in a host protein, a microbiome protein, a helminth, or a co-infecting pathogen. While the flanking and intercalated amino acids in the groove or pocket positions determine MHC binding affinity (and thus the peptide dwell time), they are hidden from the TCR. Only three variables determine the signaling directed to the TCR: whether the TCR engages the pentameric motif or not; how frequently that motif is encountered and hence the population of cognate T cells stimulated to engage it; and the dwell time of the peptide bound in the MHC groove. A precondition is that the peptide is efficiently processed by peptidases in the APC to allow excision of a peptide of appropriate length to bind in the MHC.

The second, and rather obvious, key point is that the repertoire of pentameric amino acid motifs in each recognition pattern is limited to 20^5^ or 3.2 million permutations. These may be distributed in at least three different patterns: two recognition patterns based on binding in MHC II and one pattern based on binding in MHC I (58, 60). This limits the maximal repertoire of T-cell differentiation to 9.6 million. This is entirely consistent with estimates of a human’s total T-cell count and the essential role of polyspecificity (62, 105). Given this relatively small number, it is not surprising that the large helminth proteomes we examined embodied a large percentage of the total pentameric possibilities, including the common motifs, and showed a high degree of overlap with the other proteomes and organisms examined. This mirrors findings of motif sharing among bacteria, both pathogens and commensal microbiome (57).

A further layer of variables, not addressed in this analysis, lies in the fact that multiple different clones of T cells may have TCR that bind the same TCEM peptide motif but with different affinities, and hence different dwell times, and that may trigger different cytokine outcomes (106). Higher binding affinity, consistent with higher suppressive indices, is reportedly more prone to generate IL-10 upregulation (106, 107). Binding based on less than five amino acid residues, or so-called “near neighbor” binding, further expands the possibilities for lesser binding affinities (108). The multiplicity of T-cell clones, which respond to any specific TCEM with differing binding affinities, do not change the importance of the TCEM cross-reactivity patterns but do expand the degrees of modulation possible.

The examples in Figures 3 and 4 show, for a small number of TCEM IIA motifs, that shared motifs may be embedded within many different peptides. The differing MHC GEMs, those amino acids that are directed into the MHC pockets (i.e. the non-TCEM amino acids), each determine the affinity of binding to a different set of HLA. We have focused here only on those peptides, which generate the most extreme high suppressive indices. To achieve such a score, a peptide has to be able to bind to almost all HLA alleles with high affinity. Peptides that bind with high affinity to only two or three alleles would not achieve such a high score but would have a potentially suppressive effect in individuals carrying those alleles. This reinforces two points. First, the data shown are illustrations based on the most extreme cases of epitope sharing leading to potential shared immunosuppression, when in fact a much greater span of complexity of epitope networking patterns exists across lesser degrees of binding affinity. Second, regional/ethnic population adaptation to parasites – and by the parasites to their local hosts – may be a selection function of those alleles carried by the native subpopulation binding potentially suppressive peptides with greater affinity, while those of foreign hosts do not. In addition, peptide binding to mouse, or other animal, alleles may differ from human alleles, in some cases limiting the relevance of animal models.

The analyses, even with the limitations noted, suggest that a systems approach in which the immune response is seen as a network of shared T-cell engagement may be needed to fully understand the immunomodulatory functions of helminthiasis. What is extraordinary among the helminths, which initially drew our attention, is the presence of many extremely high suppressive indices, indicative of common TCEM combined with high binding by most or many HLA alleles. In this regard, the helminths, on an individual peptide basis, far outstrip viruses and bacteria we have previously examined, where any suppressive index over 100,000 would be considered very high and where we have only in extremely rare instances observed an index of >1,000,000. Overall, this is likely due to the large helminth proteomes that increase the probability of common TCEM motifs and, when these also bind to MHC, and potentially beneficial modulatory peptides will be selected over evolutionary time.

Where shared TCEM motifs occur between helminth species, it implies that the cognate T-cell population responsive to the first helminth TCEM would also be responsive, or cross-reactive, to the same TCEM where it occurs in a second helminth. If the MHC binding of the first motif is such that it has a long dwell time and is likely to elicit a Treg response (67–70); when that same motif occurs in a second helminth and is bound by the same T-cell clones, the effect of the Treg phenotype would carry over to the response to the second helminth. Induction of relatively few Treg cells may impact the effector cells within the local microenvironment through bystander suppression (109, 110). The lengthy association of the parasite with the host, and the motif sharing among multiple parasites, would ensure a chronic presence of motifs, which have high suppressive indices, thereby fulfilling another requirement for Treg induction, repeated stimulation (111). Whereas protozoal parasites employ antigenic variation as a defensive mechanism to maintain infection (73), helminths employ the continuity of antigenic signaling to modulate the host T cell as a defense, while the surface proteins retain their invasive and other functions. It appears that relatively few Treg responders are needed to overcome a stimulatory response (67).

*Mycobacterium tuberculosis, P. falciparum*, and *H. pylori* were selected as examples of co-infections for analysis based on literature reports of interaction with helminths (7, 13, 14, 18). The widespread sharing of TCEMs among helminths, host, and the three co-infections indicates that there is potential for T-cell repertoires conditioned by exposure to a co-infecting pathogen proteome to be cross-reactive with helminths and vice versa. TCEMs provide the minimal prerequisite for T-cell polyspecificity or cross-reactivity. The shared motif counts in Table 2 are the maximum possible count of cross-reactions. This count must be further conditioned by MHC binding and peptidase processing and contemporaneous transcription. The degree of sharing is in part a function of the size of the proteome of each organism; the larger the proteome the more chance that rare motifs will be present and also that there will be more shared common motifs. However, such shared motifs can lead to mutual immunomodulatory consequences. Among the motifs that helminths share with the selected co-infections are potentially highly suppressive peptides, indicating, for example, that a Treg response elicited by *M. tuberculosis* may be shared with helminths, or vice versa. Notably, in *M. tuberculosis*, TCEM IIA matches resulting in high suppressive indices were found in 27 proteins of the PE and PE PGRS families, which have been reported to be associated with Treg induction (112, 113). In *P. falciparum*, shared high suppressive index motifs were most frequently found in rifins and in proteins annotated as conserved plasmodium proteins. Rifins are a family of proteins expressed on *P. falciparum*-infected erythrocytes, which have been implicated in immune evasion through binding to immune inhibitory receptors and the development of severe malaria (114, 115). Less sharing was seen in *H. pylori*, which has a smaller proteome and where the interaction with helminths may not be dependent on immune mechanisms (18).

A networking model in which a helminth infection creates a Treg cell-rich microenvironment and simultaneously causes chronic tissue damage, and thus a high rate of host cell division prone to mutations, may also create an environment tolerant to the progression of neoplasia. Treg cells have been demonstrated to be increased in *Opisthorchis* infections (40) and may lead to impaired response to neoepitopes. While multiple mechanisms may trigger carcinogenesis, such a model could explain the emergence of cholangiocarcinoma following prolonged infection with *Clonorchis* or *Opisthorchis* (15, 18). Conversely, the increase in autoimmune, inflammatory, and allergic disease observed in the absence of helminths, or following anthelminthic application, would be consistent with removal of the peptides driving an immunosuppressive Treg-dominated network.

Helminth proteins contain a large number of homopolymers of amino acids along with repetitious simple amino acid motifs. Only a few homopolymers (glycines, leucines, serines, threonines, and tyrosines) contribute to the counts of higher frequency TCEMs; of these, only leucines and tyrosines result in fairly high binding affinity for multiple alleles. While such repeats are a more common feature of eukaryotes than prokaryotes (116) and may offer some selective advantages (117), they do not emerge as a driver of very high suppressive indices in the helminths examined. However, their presence does raise the question of whether multiple occurrences, especially of leucine or tyrosine observed in *L. loa*, might have an additive suppressive effect.

We omitted from consideration proteins under 50 amino acids in length. Some helminth proteomes included a large number of small open reading frames (sORFs). Whether sORFs which generate peptides large enough to bind MHCs may also contribute to the overall epitope landscape is unknown (118, 119). Given species differences in the curation of proteins for inclusion, a comparative analysis is not possible.

In addressing networking as a factor in immunomodulation, we have focused here on the most common TCEM and those that are shared by many helminths. We also noted that a very small percentage of TCEM motifs are unique to any single helminth among those evaluated; these may or may not be motifs rarely found in the immunoglobulinome but are unlikely to be among the most common motifs. Figure 1 reflects the sharing patterns of motifs among helminth species. There is a range of less common motifs, which also have sharing patterns among the species (as reflected by the grey and red tiles in Figure 1). These are motifs that may elicit immune stimulation and potentially have utility in a vaccine. The challenge in designing a vaccine comprising T-cell epitopes is to identify proteins comprising conserved stimulatory epitopes, appropriately processed by cathepsins for MHC presentation, which are not negated by an immunosuppressive motif (120).

It is likely that many mechanisms are contributing simultaneously to immunomodulation. The proteins previously reported (24, 27, 28, 37) may have a primary or secondary role in immune regulation. The cystatins, which have been reported to play an immunomodulatory role in several parasites (121–123), would likely inhibit cathepsin function and, hence, peptide excision for presentation on the MHC. Others may act through the induction of Treg cells or as a downstream consequence of such regulation. Among the previously reported immunomodulators that we analyzed, only *T. spiralis* p53 was found to have a suppressive index over 300,000. Several others had suppressive indices below this level, indicating that they might have a suppressive effect in individuals of certain alleles, or when present in large amounts; the major egg antigen of *S. mansoni* is one example. However, it is important to keep in mind that several of the proteins previously examined for their immunomodulatory role are from helminths that are not human pathogens and they have been examined in animal models, so our analysis of suppressive index based on human alleles examines those out of the host context in which they were tested.

There has been increasing interest in exploiting the immunomodulatory effects of helminths (23, 26), by administering low level live parasite infections, extracts, or recombinant proteins (24, 25, 31). The TCEM networking model put forth here offers an approach to defining predicted immunomodulatory peptides, which have benefited from selection through an evolutionary filter and which may then be tested in a therapeutic model at less risk than a live parasite.

Given the lack of annotation of many individual proteins in the helminth proteomes, and the recognition that we have only worked with one isolate of each species, we have not focused here on highlighting the particular potential highly suppressive peptides for each parasite. Rather we have addressed this as a conceptual model. For every motif example for which we show an unambiguous predicted outcome for most HLA, there are many more subtle effects based on lower suppressive indices or particular host HLAs. The systems approach we have taken suggests that the immune response to a given parasite cannot be considered in isolation but should be seen as a part of an epitope ecosystem, or microenvironment, in which the potentially highly suppressive peptides in the helminths network through their common T-cell receptor recognition signals with T-cell epitopes in self proteins, microbiome, in other helminths and in taxonomically unrelated pathogens. It indicates the complexity of T-cell interactions, which may have allowed helminths to evolve to select for the peptides that drive an immunosuppressive repertoire of T cells. Such a repertoire would favor long-term coexistence with the host, or an immunogenetic subpopulation thereof, and facilitate polyparasitism and co-infections. This approach suggests that a different paradigm, in which epitope networking is considered, can contribute to understanding helminth associated immunomodulation. It is a paradigm, which is both very simple in conception and profoundly complex to test through a reductionist approach.

## Author Contributions

RB designed the algorithms, EH planned the study and performed the analyses, and EH and RB wrote the article.

## Conflict of Interest Statement

Both authors are employees and equity holders in ioGenetics LLC, the parent company of EigenBio LLC. The reviewer PT and handling Editor declared their shared affiliation.

## Abbreviations

APC, antigen-presenting cell; FC, frequency category; GEM, groove-exposed motif; HLA, human leucocyte allele; MHC, major histocompatibility complex; pMHC, peptide MHC; sORF, small open reading frame; TCEM, T-cell-exposed motif; TMH, transmembrane helix.
