# Supplementary material for: A Role for Epitope Networking in Immunomodulation by Helminths
Source: Front Immunol. 2018 Jul 31;9:1763. doi: 10.3389/fimmu.2018.01763 (PMC6079203; doi:10.3389/fimmu.2018.01763)
Supplement: Supplementary file 1 [file Data_Sheet_1.PDF]

Supplemental Materials

A role for epitope networking in immunomodulation by helminths.

E. Jane Homan, Robert D. Bremel

Supplemental Table S1: Reported immunomodulatory proteins evaluated.

Supplemental Table S2: Sharing of TCEM IIB and TCEM I motifs between helminths, human immunoglobulinome, gastrointestinal microbiome, and proteome.

Supplemental Table S3: Motif sharing with coinfections.

Supplemental Figure S1: Illustrations of suppressive indices.

Supplemental Figure S2: Bivariate plots of multiple helminth species.

Supplemental Figure S3: Sequences of *Onchocerca volvulus* proteins highlighted in Figure 2

Supplemental Table I: Proteins with previously reported immunomodulatory role.

Protein	function	Highest MHC I suppressive index noted	Highest MHC II suppressive index noted	Murine allele binding*	Reference
Acanthocheilonema vitae cystatin AvCystatin gi 867675	cystatin	8,632	978		#
Acanthocheilonema vitae ES62 gi 3386478		2,668	7,327		##
Ancylostoma caninum, tissue inhibitor of metalloproteases Ac-TMP2 (also Ac-AIP-2) gi 170295877	protease	375	771		(1, 2)
Anisakis simplex AsMIF gi 120564780	macrophage inhibition factor	2,056	1) 54,718<br>2) 35,096	1) neither mouse<br>2) neither mouse	#
Brugia malayi Bm CPI2 gi 5759313	pepsin inhibitor	842	18,451		#
Clonorchis sinensis Annexin B rCsANXB30 gi 358341210	Binding protein	76,386	3,231	neither mouse	#
Clonorchis. siniensis Type 1 cystatin CsStefin-1 gi 150404780	cystatin	478	1,899		#
Dirofilaria immitis rDIAg gi 1663728	polyprotein	7,653	884		#
Fasciola hepatica FhHDM1 gi 325513923	cathelicidin like	1,308	4,722		#
Fasciola hepatica glutathione transferase rFheGST gi114797025	antioxidant	231	1,601		#
Fasciola hepatica protease cathepsin L rFheCL1 gi 116488416	protease	2,313	1) 81,960<br>2) 33,203	1) neither mouse<br>2) H2-I-Ab and H2-I-Ad	#
Haemonchus contortus rHCo-gal gi 2687413	lectin	1) 84,031	2) 40,852	1) H2-Db<br>2) H2-1-Ab and H2-1-Ad	#
Heligmosomoides polygyrus cystatin rHp-CPI gi432134292	cystatin	607	175		#
Nippostrongylus brasilensis Nippostatin gi 14278939	cystatin	375	4,353		##
Schistosoma mansoni cathepsin SmB1 gi22531387	protease	821	24,648	H2-1-Ad	#

Schistosoma mansoni Sm29 gi 4090941	membrane bound glycoprotein	546	1,348		#
Schistosoma mansoni SmKI XM_018795470.1	serine protease inhibitor	2,852	2,592		(3)
*Schistosoma mansoni **ω**-1 gi 129359*	egg antigen	1,132	57,457	H2-1-Ab	##
Strongyloides ratti Sra-MIF gi 198448303	macrophage inhibition factor	257	12,437		#
Toxocara leonina Galectin 9 gi 1187200208	Lectin	64,981	14,380	H2-Db	#
Trichinella spiralis glycoprotein rTSP53 gi 339252784	glycoprotein	8,553	305,422	H2 1 Ab	#

*For peptides exhibiting suppressive indices in excess of 20,000 we note which murine alleles are bound at >0.75 standard deviation units below the mean for that protein (moderate binding); these would therefore likely be suppressive in these mice (BALB/c: Kd, Dd, H1-I-Ad; C57BL6: Kb, Db, H1-IAb)

Reviewed in (4)

Reviewed in (5)

1. Navarro S, Pickering DA, Ferreira IB, Jones L, Ryan S, Troy S, et al. Hookworm recombinant protein promotes regulatory T cell responses that suppress experimental asthma. *Science translational medicine* (2016) **8**(362):362ra143. doi: 10.1126/scitranslmed.aaf8807. PubMed PMID: 27797959.
2. Cantacessi C, Hofmann A, Pickering D, Navarro S, Mitreva M, Loukas A. TIMPs of parasitic helminths - a large-scale analysis of high-throughput sequence datasets. *Parasites & vectors* (2013) **6**:156. doi: 10.1186/1756-3305-6-156. PubMed PMID: 23721526; PubMed Central PMCID: PMCPMC3679795.
3. Morais SB, Figueiredo BC, Assis NRG, Alvarenga DM, de Magalhaes MTQ, Ferreira RS, et al. Schistosoma mansoni SmKI-1 serine protease inhibitor binds to elastase and impairs neutrophil function and inflammation. *PLoS pathogens* (2018) **14**(2):e1006870. doi: 10.1371/journal.ppat.1006870. PubMed PMID: 29425229.
4. Nascimento Santos L, Carvalho Pacheco LG, Silva Pinheiro C, Alcantara-Neves NM. Recombinant proteins of helminths with immunoregulatory properties and their possible therapeutic use. *Acta tropica* (2017) **166**:202-11. doi: 10.1016/j.actatropica.2016.11.016. PubMed PMID: 27871775.
5. Maizels RM, McSorley HJ. Regulation of the host immune system by helminth parasites. *J Allergy Clin Immunol* (2016) **138**(3):666-75. doi: 10.1016/j.jaci.2016.07.007. PubMed PMID: 27476889; PubMed Central PMCID: PMCPMC5010150.
6. Bremel RD, Homan EJ. Frequency Patterns of T-Cell Exposed Amino Acid Motifs in Immunoglobulin Heavy Chain Peptides Presented by MHCs. *Frontiers in immunology* (2014) **5**:541. doi: 10.3389/fimmu.2014.00541. PubMed PMID: 25389426; PubMed Central PMCID: PMC4211557.

Supplemental Table 2: Sharing of TCEM IIB and TCEM I motifs between helminths, human immunoglobulinome, gastrointestinal microbiome, and human proteome

	Total TCEM IIB motifs	TCEM IIB as percent of 3.2 million	TCEM IIB shared with GI microbiome	% TCEM IIB shared with GI microbiome	TCEM IIB Shared with human proteome	% TCEM IIB shared with human proteome	TCEM IIB unique to helminth species	Unique TCEM IIB as % of species total
Immunoglobulinome	3,200,000	100.00%						
GI Microbiome	2,927,585	91.49%						
Human proteome #1	2,420,644	75.65%						
Total motifs in 17 helminths	3,103,783	96.99%	3,103,391	99.99%	2,778,161	89.51%		
TCEM IIB common to all 17 species	419,378							
Ancylostoma ceylanicum	2,286,461	71.45%	2,200,074	96.22%	1,939,224	84.81%	12,666	0.55%
Ancylostoma duodenale	1,850,934	57.84%	1,798,214	97.15%	1,617,375	87.38%	4,151	0.22%
Necator americanus	1,850,923	57.84%	1,799,187	97.20%	1,618,459	87.44%	5,301	0.29%
Ascaris lumbricoides	2,081,984	65.06%	2,013,813	96.73%	1,793,032	86.12%	13,219	0.63%
Brugia malayi	1,842,257	57.57%	1,789,668	97.15%	1,604,924	87.12%	2,439	0.13%
Loa loa	1,803,177	56.35%	1,753,506	97.25%	1,577,594	87.49%	3,553	0.20%
Onchocerca volvulus	1,860,756	58.15%	1,807,112	97.12%	1,618,075	86.96%	4,888	0.26%
Wucheria bancrofti	1,743,181	54.47%	1,696,340	97.31%	1,526,655	87.58%	2,049	0.12%
Trichinella spiralis	1,883,947	58.87%	1,823,036	96.77%	1,634,394	86.75%	12,165	0.65%
Trichuris trichura	1,720,825	53.78%	1,674,730	97.32%	1,513,596	87.96%	9,118	0.53%
Clonorchis sinensis	2,086,265	65.20%	2,014,652	96.57%	1,801,115	86.33%	7,477	0.36%
Opisthorchis viverrini	2,038,194	63.69%	1,969,811	96.64%	1,767,509	86.72%	6,587	0.32%
Fasciola hepatica	1,922,829	60.09%	1,862,106	96.84%	1,677,936	87.26%	10,611	0.55%
Schistosoma mansoni	1,827,249	57.10%	1,773,498	97.06%	1,593,181	87.19%	9,043	0.49%
Diphyllobothrium latum	1,595,794	49.87%	1,556,727	97.55%	1,422,817	89.16%	6,496	0.41%
Echinococcus granulosus	1,793,953	56.06%	1,743,418	97.18%	1,583,268	88.26%	4,538	0.25%
Taenia solium	1,806,756	56.46%	1,755,533	97.16%	1,593,398	88.19%	4,751	0.26%

	Total TCEM I motifs	TCEM I as percent of 3.2 million	TCEM I shared with GI microbiome	% TCEM I shared with GI microbiome	TCEM I Shared with human proteome	% TCEM I shared with human proteome	TCEM I unique to helminth species	Unique TCEM I as % of species total
Immunoglobulinome	3,200,000	100.00%						
GI Microbiome	2,906,343	90.82%						
Human proteome #1	2,389,984	74.69%						
Total motifs in 17 helminths	3,096,778	96.77%	3,096,166	99.98%	3,093,873	99.30%		
TCEM I common to all 17 species	414,414							
Ancylostoma ceylanicum	2,266,982	70.84%	2,172,853	95.85%	1,907,133	84.13%	13,230	0.58%
Ancylostoma duodenale	1,831,139	57.22%	1,773,188	96.84%	1,588,064	86.73%	4,081	0.22%
Necator americanus	1,833,134	57.29%	1,776,023	96.88%	1,591,146	86.80%	5,616	0.31%
Ascaris lumbricoides	2,056,872	64.28%	1,981,951	96.36%	1,756,664	85.40%	13,493	0.66%
Brugia malayi	1,819,909	56.87%	1,761,657	96.80%	1,573,047	86.44%	2,366	0.13%
Loa loa	1,781,836	55.68%	1,727,123	96.93%	1,547,375	86.84%	3,544	0.20%
Onchocerca volvulus	1,839,531	57.49%	1,780,652	96.80%	1,587,409	86.29%	4,944	0.27%
Wucheria bancrofti	1,723,239	53.85%	1,671,227	96.98%	1,497,899	86.92%	2,040	0.12%
Trichinella spiralis	1,852,416	57.89%	1,786,078	96.42%	1,595,161	86.11%	12,613	0.68%
Trichuris trichura	1,703,295	53.23%	1,652,513	97.02%	1,486,906	87.30%	9,529	0.56%
Clonorchis sinensis	2,068,485	64.64%	1,989,636	96.19%	1,770,875	85.61%	8,093	0.39%
Opisthorchis viverrini	2,019,860	63.12%	1,944,270	96.26%	1,737,111	86.00%	6,969	0.35%
Fasciola hepatica	1,905,879	59.56%	1,838,899	96.49%	1,649,642	86.56%	11,445	0.60%
Schistosoma mansoni	1,808,191	56.51%	1,749,291	96.74%	1,565,167	86.56%	9,412	0.52%
Diphyllobothrium latum	1,580,028	49.38%	1,536,089	97.22%	1,399,827	88.60%	6,796	0.43%
Echinococcus granulosus	1,775,514	55.48%	1,719,115	96.82%	1,555,786	87.62%	4,806	0.27%
Taenia solium	1,788,809	55.90%	1,731,986	96.82%	1,566,151	87.55%	5,067	0.28%

#1 Human proteome excluding immunoglobulins, all isoforms, 88,000 proteins

Supplemental Table 3: Motif sharing with coinfections

Helminth	TCEM IA Percent X *P.falciparum*	TCEM IIA Percent X *P.falciparum*	TCEM IIB Percent X *P.falciparum*	TCEM I Percent X *M.tuberculosis*	TCEM IIA Percent X *M.tuberculosis*	TCEM IIB Percent X *M.tuberculosis*	TCEM I Percent X *H. pylori*	TCEM IIA Percent X *H. pylori*	TCEM IIB Percent X *H. pylori*
Ancylostoma ceylanicum	45.16%	45.70%	45.80%	26.30%	26.49%	45.80%	14.61%	14.85%	14.96%
Ancylostoma duodenale	47.65%	48.23%	48.29%	29.18%	29.37%	48.29%	16.25%	16.53%	16.65%
Necator americanus	48.07%	48.59%	48.68%	29.08%	29.30%	48.68%	16.31%	16.59%	16.71%
Ascaris lumbricoides	46.60%	47.15%	47.24%	27.58%	27.74%	47.24%	15.42%	15.66%	15.76%
Brugia malayi	49.74%	50.30%	50.41%	28.11%	28.35%	50.41%	16.50%	16.77%	16.89%
Loa loa	49.70%	50.23%	50.37%	28.62%	28.85%	50.37%	16.65%	16.94%	17.06%
Onchocerca volvulus	49.80%	50.32%	50.44%	27.91%	28.13%	50.44%	16.45%	16.74%	16.85%
Wucheria bancrofti	50.53%	51.08%	51.22%	28.61%	28.85%	51.22%	16.94%	17.22%	17.33%
Trichinella spiralis	48.49%	49.05%	49.13%	27.89%	28.06%	49.13%	16.08%	16.38%	16.52%
Trichuris trichura	48.36%	48.97%	57.14%	29.74%	29.91%	57.14%	16.76%	17.05%	20.01%
Clonorchis sinensis	45.53%	46.19%	46.30%	27.74%	27.94%	46.30%	15.15%	15.42%	15.54%
Opisthorchis viverrini	45.68%	46.31%	46.41%	28.12%	28.31%	46.41%	15.30%	15.59%	15.69%
Fasciola hepatica	46.39%	47.02%	47.20%	28.82%	29.01%	47.20%	15.71%	16.00%	16.12%
Schistosoma mansoni	50.78%	51.31%	51.43%	27.69%	27.91%	51.43%	16.48%	16.76%	16.88%
Diphyllobothrium latum	48.66%	49.20%	49.25%	31.21%	31.41%	49.25%	17.25%	17.55%	17.68%
Echinococcus granulosus	47.50%	48.05%	48.14%	29.79%	30.02%	48.14%	16.45%	16.70%	16.80%
Taenia solium	47.33%	47.88%	48.01%	29.79%	29.98%	48.01%	16.38%	16.65%	16.76%

Plasmodium falciparum 3D7; *Mycobacterium tuberculosis* H37Rv; *Helicobacter pylori* strain 26695

Supplemental Figure S1: Suppressive Indices

The suppressive index is a predictive metric for the potential number of cognate T cell collisions with pMHC displaying a particular TCEM. Its purpose is to enable population-wide epitope ecosystem estimates. Conceptually, each doubling of a TCEM frequency (log2 FC reduction) will lead to a doubling of cognate T cell collisions with a pMHC containing a peptide of a particular FC. Having a peptide bound by a number of different MHC alleles and presented as a pMHC complex in a population will lead to that population exhibiting a common phenotype – e.g. susceptibility to T cell suppression by the source protein in a parasite. FC 16 ($1/2^{16}$ clonotypes) is set as the baseline because it corresponds to the number of IGHV previously computed (6) and which contain the complete VDJ regions with all three CDRs. It is not intended to imply the presence of a single cognate T cell clonotype in an individual's repertoire.

Suppressive Index

$$SI = -\sum_{i=1}^{N} (nIC50_A)_i * CT_A$$

$nIC50_A$ = standardized binding affinity estimate for MHC allele A. Note that standardized high affinities are below the mean and thus negative

N = total number of alleles

CT_A = number of cognate T cell for pMHC allele A

FC	CTA	Suppressive Index @ -2 stdev (N=10 allleles)
1	32,768	655,360
2	16,384	327,680
3	8,192	163,840
4	4,096	81,920
5	2,048	40,960
6	1,024	20,480
7	512	10,240
8	256	5,120
9	128	2,560
10	64	1,280
11	32	640
12	16	320
13	8	160
14	4	80
15	2	40
16	1	20
17	0.5	10
18	0.25	5
19	0.125	2.5
20	0.0625	1.25
21	0.03125	0.625
22	0.015625	0.3125
23	0.0078125	0.15625
24	0.00390625	0.078125

The two components – TCEM frequency and MHC binding affinity are independent variables -one stochastic, the other determined by the genetics of the host. In the case of MHC II two pentamer exposed motif frames of the peptide are recognized (~2,3~5~7,8~ or -1~~3~5~7,8~ respectively where ~ is the intervening positions, relative to the central nonamer core of a longer bound peptide) and so a 15mer may carry a common motif in both frames, one, or neither configurations. Based on its size and turnover we use the immunoglobulin variable region as a reference scale of common vs rare motifs (see reference 65) now supported by analysis of over 40 million variable region sequences from human B cell repertoires. The occurrence of TCEM pentamers is stochastic and so occurrence of the common motifs is related to proteome size. Approximately 0.02% of the TCEM IIa motifs in any proteome are found more commonly than 1 in 8 immunoglobulin variable regions (FC1+FC2+FC3) irrespective of the protein origin (virus, bacteria, fungi, parasites). The second variable is then the binding to MHC determined by host genetics and based on the flanking pocket or groove exposed amino acid motifs. The following figures show how number of alleles and their binding affinity impact the suppressive index as a population indicator. For simplicity, we have focused on TCEM IIA and assumed that both frames have the same TCEM frequency score.

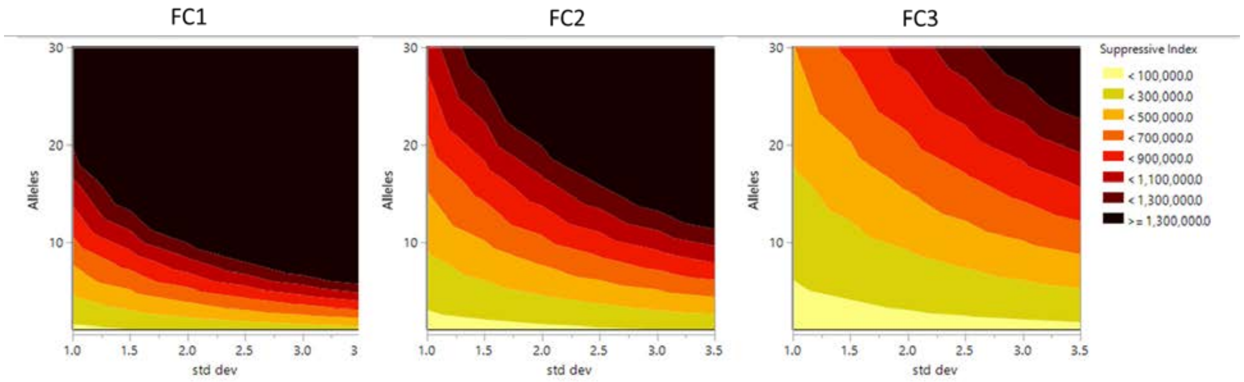

The threshold we used in our analysis was 300,000, an empirically determined rare occurrence. The graphics show how a suppressive index of this magnitude can be achieved in a multivariate context. A suppressive index of >300,000 (as indicated in the orange through black zones) could, for instance, be the result of 3 alleles binding a FC1 motif in both frames with a relative affinity of -1 standard deviation units or by 10 alleles binding in both frames with an affinity of -2 standard deviation units.

Supplemental Figure S2: Proteins with high content of common TCEM Motifs. Each dot represents one protein of the species indicated. X axis shows size of the protein ($\log_{10}$ amino acid number). Y axis shows number of TCEM IIA motifs of higher than FC10, i.e. occurring more often than the mean in an immunoglobulinome reference database. Proteins shown in blue are secreted and surface proteins.

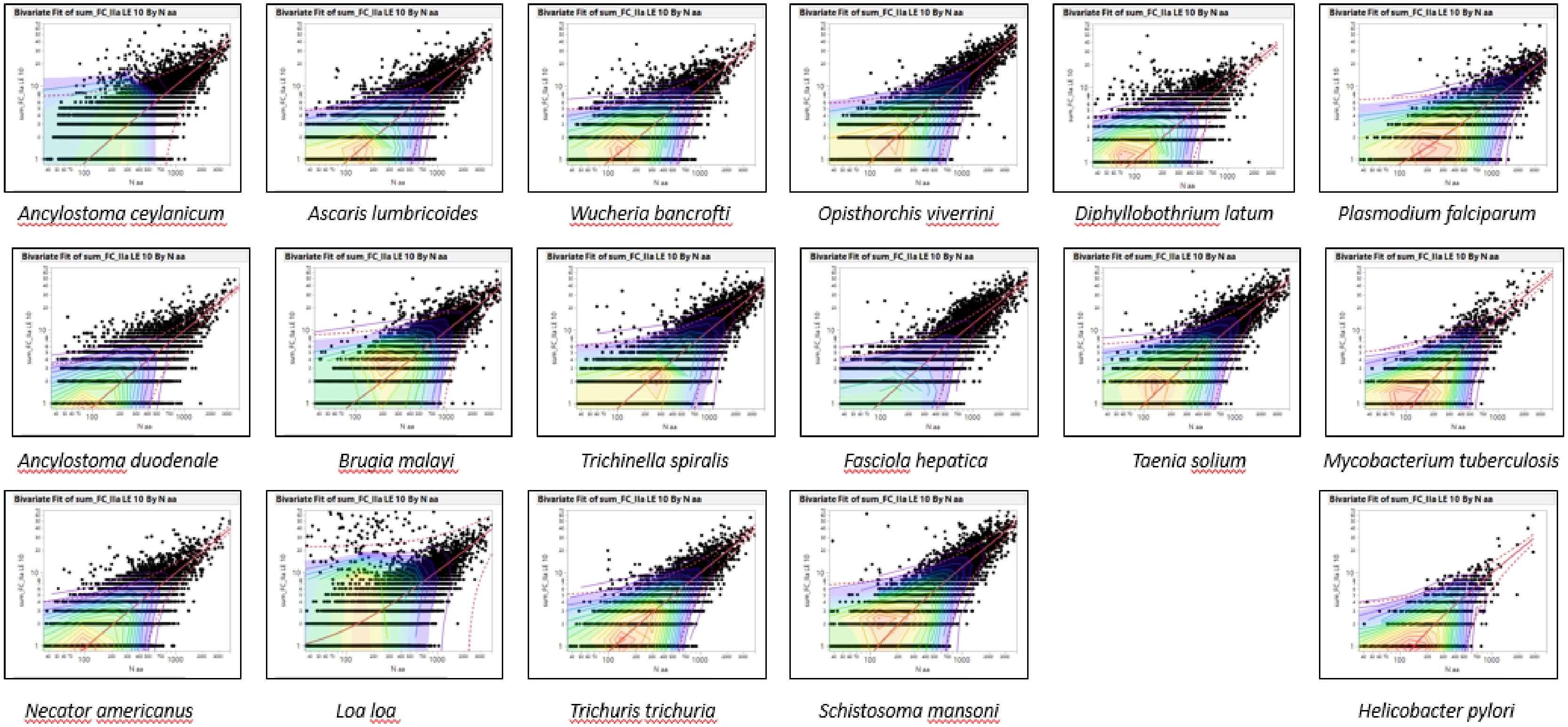

Supplemental Figure S3: Sequences of *Onchocerca volvulus* proteins highlighted in Figure 2

OVOC1831
MQHDKNATETAGVSLSPITPYDSFVQQQQQQQQQQQQQQQQQRQQQQQQEQQQQQQPSSSSSVLPTRMISSKSFELLNSDDGDSSEVQDSLQQQRQQQQHQNDTTQKLLAQLTSTISSGQVSHAIIPQQIYMATAAAEEAKLLETRKQQ
LLQKLQQQLNSSKISANGTANTTVAAQLWLQQSCAATAALQQQQQQQLISKTNQNQSSTVLSNHSTTANLISETEQQIITCQQDIQQQQQQTSFLTSDYEQFVQNGDLTQLKDQNELQSLSNSKTTTVQQQYHHPMVHAGDGSSAFRQPQRQ
QQYLFDQNTKIQQDLATTVIETVTRSCGDDASSFAVPSSSSSSSSSSSSSSSSSTTSSLSNNSSFISSLSRSHVISVKQSYHNEKTDGTEPKLIISDIEMTDDCNKTDKTIESYPVSKIADKEEGQSLLKIENRGSEIIGISTYATIGAMIHMDNDEFVEKTD
NAGEEPRMKSILNFVDGEVEIDVEHKESDENEKNEKRILDNDDPAGSGSVNDNETRPLVNKMESPEIHGTRIEAPLEQTRFTAMRIEERILNEKLCPSTASSTATTSGSMVISSPCHRLVSQSVSVAPSLRSESVSSTPSSSTFCRAPGLGPVNTTETQQS
GTPLPLVCNICGFSCNSKFHYNSHMNTHGDHQCTMCDYTSRTEGRLKKHMRDSHTVEEQLAAGLDVEQALTGSASTSSTPVTRIATTPVDGSNLATSMASVLEAANLAVAAQAAAQAAAASCSSSSCSGPTTSASDSSSGLSLPSGSGTPPSSSDIIS
SSSFSAVATSISGLVPSALDQIRAFTENPSVLPDLSSANLATALISQGLLSSQAFQESIKSINSDEASVSPVNGDKPRQCSSKPKTYKCKQCSHISTSKEEQWTHARTHIPIEKQLGCTRCGFVTEYKHHLEYHLRNHMGSKPFHCKKCAYSCVNKSMLNS
HMKSHTNIYQFRCRDCTYATKYCHSLKLHLRKYNHNRATDITDSAVGIAINATASTSNLCNGSTQAVTENDDTTTMRRLSESLAVQSVDCSSPEPLPTTSTMAQEITAALGMTPVVTSNSLNIASHLLFRQQHQVQMEEMELLLRLKNMTGMNPGA
MLGISLQCPVCSYQAINQGDSLRHQLGHLIGPNGENPLIPLYNIFGVPQPSNETAFESSSNINEQMEQQDEEMEASKVEVDDFISGAEVDEKPSTADSEDHNMSDISGESQDSPSSGKSSIDEGSSGMEQESDKGAKKRKAGLKLNQIAARLQEKNS
PENPSTSDSINDSKYEELLLSRMKENPLSEMSGTSLGITLESQEAPSISPLPSSTTSLVPQQPQLVPSLGMPNNLMNLSNLNIFQQACIAHMQDMERKLHILEMWRFSCAHCKMAFQDEPLYHIHMGYHGYENPFKCNRCGQACSDFLTFNLHLLQG
KH

OVOC6031
MNIEEILPELFGEKRVYYCQRCLNHGLEVKRKNHKSECAYRFCICNDCQMVDKRRELNSRLLQIESISSPPPPLPAPPPLPLALPPTPLLSLSLSSSSTSSSSSSSSSSSSSSSSSSSSSSSSSSSSSSLSSLSQSLSNNNFLTHVPLLPDMNLFSPTPEQAISPL
AVKGKERRPNCQRCAQHSVLTRLKGHKRCCPFRDCPCAKCQVVQERQKLMADQIKLRRRQKKQKNLDALNHSNNLRRTIGFSKQQGPVFSGCLKCAQQVLAYQQILTFIDPNATLQPSAFLAILAACPHNNFNNHNNSNNINNSINNSFNDNK
NDNYKT

OVOC5909
MNIKKGIAFLFIIASSSYAMPKSYYSSRTTISGPLCSLVHWIYEKIFGLQWIQPMRMSWIPKSSTSSSTSIFSTRATSARSPTTTVTIESSSTTSPTFPITTEPSSTIPAIKPFSSQNCSCFKLFYQRAVTTSPTATVTTESSSTPPTSTVTIESTSTISPTITLATESSS
AISPTTTVATESSSTTTVTTEPSSTIPAIKPFSLQNCSCFKLLNQRAVTTFPTSTVTIESSSTTSPTTTVTAESSSATSPTTIAMTISSSTTSPTSTITTESSSITSPTKTVTSESSKTTPITESSATFPTTIAMTKSSSTTSPTSTITTESSSITSPTKTVTSESSKTTPITES
SATSPTTIAMTKSSSTTSPTSTVKTEFSSTVSPTSTVATESSSTTTVITESPSITSPKSRITTESPSTTSSTITVTTEKSPTTRVTTESSSTTSPTSTVTTEFSTTLASAVATESSSTISPTSTVTTESSTISTTSTVKTDSSPTTPTSTVTTESPTTSPKSTVATESSPTTT
VTAEKSPTTTVATESSSTISPTSTLTTESSSTTSPTSTVTTEFLTTLTSTVATESSSKISTTTTLTTDSSSTAFPKSTVATESPPTTSPKSTVATESPSTITVTTEKSPTTTVTTESPSTTTVITEKSPTTTVATESSSTTSPKSTEATESPSTTTVTTEKSPTTTAATEAS
STISPTSTVTTESSSTTSPTTTVTSESSSTTTVTTESSPTTTAKTKSSSTTPTPAVTESSTISPTSTVTTESSSRTTPTTTAATKSLTTSPTTTVTIGFSSTTSATTKASSTTSDLARILQQLSLLIRDLQTSLH
